# Dual-modality ultrasound radiomics model for classifying diabetic peripheral neuropathy in type 2 diabetes: a multicenter prospective study

**DOI:** 10.3389/fendo.2026.1900881

**Published:** 2026-08-13

**Authors:** Rong Xiao, Wanyan Li, Wenqian Qiu, Chenglong Zhang, Enfa Zhao, Tingting Xie, Danqing He

**Affiliations:** 1 Department of Ultrasound, The First Affiliated Hospital of Anhui Medical University, Hefei, Anhui, China; 2 Department of Ultrasound, Linquan County People’s Hospital, Fuyang, Anhui, China; 3 Department of Ultrasound, The Hefei BOE Hospital, Hefei, Anhui, China; 4 Department of Ultrasound, The First Affiliated Hospital of Anhui Medical University, South Zone Branch, Hefei, Anhui, China

**Keywords:** diabetic peripheral neuropathy, machine learning, radiomics, SHapley additive explanations, shear wave elastography

## Abstract

**Objective:**

Diabetic peripheral neuropathy (DPN) is a prevalent and disabling complication of type 2 diabetes mellitus (T2DM), yet detection remains constrained by limited accessibility of nerve conduction studies. This study developed and validated a dual-modality ultrasound radiomics model for individualized DPN classification.

**Methods:**

In total, 253 T2DM patients from three centers were prospectively enrolled between June 2025 and February 2026, allocated to training (n = 122), internal test (n = 53), and external validation (n = 78) cohorts. Radiomics features were extracted from longitudinal B-mode ultrasound and shear wave elastography images of the tibial nerve. After reproducibility filtering, batch-effect correction, and elastic-net feature selection, four machine learning algorithms were compared and the best-performing was used to construct modality-specific radiomics scores (Rad-scores). A combined model integrating Rad-scores with independently associated clinical factors was developed. Discrimination, calibration, clinical usefulness, and SHapley Additive exPlanations (SHAP) interpretability were evaluated.

**Results:**

Multivariable analysis identified diabetes duration and minimum elastic modulus as independently associated factors, with both Rad-scores significantly associated with DPN status. The combined model demonstrated good discrimination across all cohorts (AUC 0.892 [95% CI 0.828–0.950], 0.824 [0.696–0.925], and 0.838 [0.735–0.926]), outperforming all other models (*p* < 0.05), with adequate calibration in both training and internal test cohorts (*p* > 0.05). Decision curve analysis confirmed clinical benefit. SHAP analysis identified diabetes duration as the most influential variable, followed by the shear wave elastography Rad-score.

**Conclusions:**

The combined model demonstrates favorable diagnostic performance for individualized DPN risk stratification and holds promise as a noninvasive complement to nerve conduction.

## 1 Introduction

Affecting nearly half of patients with type 2 diabetes mellitus (T2DM), diabetic peripheral neuropathy (DPN) frequently culminates in diabetic foot ulceration and lower limb amputation (1, 2), and represents a growing global burden given that the number of people living with diabetes is expected to surge from 537 million in 2021 to 783 million by 2045 (3). Early and accurate identification of DPN is therefore critical; however, the diagnostic gold standard, nerve conduction studies (NCS), depends on specialized equipment, operator expertise, and patient tolerance, substantially limiting its accessibility in routine screening and contributing to widespread underdiagnosis (4, 5).

High-resolution ultrasound has emerged as a promising complementary modality, enabling direct visualization of characteristic morphological changes in DPN-affected nerves including increased cross-sectional area and disrupted echo architecture (6, 7). Among the peripheral nerves affected by DPN, the sural nerve is involved early but its small caliber and anatomical variability limit its suitability for reproducible ultrasound imaging; by contrast, the tibial nerve, with its superficial course and relatively fixed position at the medial malleolus, is similarly involved early in the length-dependent progression of DPN and is more amenable to reproducible high-resolution visualization. More recently, shear wave elastography (SWE) has demonstrated the ability to quantify tibial nerve stiffness, with elevated elastic moduli reflecting endoneurial fibrosis (8). Nevertheless, single quantitative SWE parameters such as Emin and Emean are subject to inter-device variability and operator dependence, capturing only macroscopic mean stiffness while failing to characterize the spatial heterogeneity of elastic modulus distribution within nerve fascicles (9).

By mining high-dimensional quantitative imaging features beyond the perceptual limits of human vision, radiomics enables objective tissue characterization beyond visual assessment (10, 11). When integrated with machine learning algorithms, radiomics-derived features can be systematically combined to construct robust diagnostic models capturing complex nonlinear relationships among imaging biomarkers (12, 13). Although B-mode ultrasound (BMUS) radiomics has achieved diagnostic AUC ranging from 0.87 to 0.95 in recent multicenter DPN diagnostic studies (14, 15), these investigations extracted features exclusively from gray-scale morphological images, failing to capture biomechanical alterations encoded in elastographic imaging. Furthermore, while SWE-derived scalar elasticity parameters have been incorporated as individual variables in DPN models (16), high-dimensional SWE radiomics features and their integration with BMUS radiomics within a unified dual-modality framework have not been investigated, representing a critical methodological gap in current clinical DPN management.

We hypothesized that such a dual-modality framework would outperform single-modality radiomics and the clinical model for individualized DPN classification. Accordingly, the present multicenter prospective cross-sectional diagnostic study developed and validated a model integrating BMUS radiomics, SWE radiomics, quantitative elasticity indices, and independently associated clinical factors across three centers. To address the inherent black-box limitation of machine learning, SHapley Additive exPlanations (SHAP) analysis was additionally applied to provide both global and individual-level interpretability of model outputs, thereby enhancing the clinical transparency of the proposed framework (17).

## 2 Materials and methods

### 2.1 Study population

This prospective cross-sectional diagnostic study adhered to the principles of the Declaration of Helsinki and received ethical approval from the Institutional Review Board of the First Affiliated Hospital of Anhui Medical University (approval No. PJ2025-02-43), and written informed consent was obtained from all participants. Between June 2025 and February 2026, patients with T2DM were prospectively and consecutively enrolled from three centers: the First Affiliated Hospital of Anhui Medical University (Center 1), its South Zone Branch (Center 2), and Linquan County People’s Hospital (Center 3).

Eligible patients were required to meet all of the following criteria: (i) T2DM diagnosed based on World Health Organization (WHO) criteria; (ii) age ≥ 18 years; and (iii) willingness to undergo a research-specific lower-extremity ultrasound examination. Patients were excluded if they had: (i) peripheral neuropathy attributable to hereditary, metabolic, or inflammatory etiologies other than diabetes; (ii) lower extremity skin lesions or active infection; (iii) a history of lower extremity surgery or trauma; or (iv) poor-quality ultrasound images or incomplete ultrasound or clinical data.

**Figure 1:**
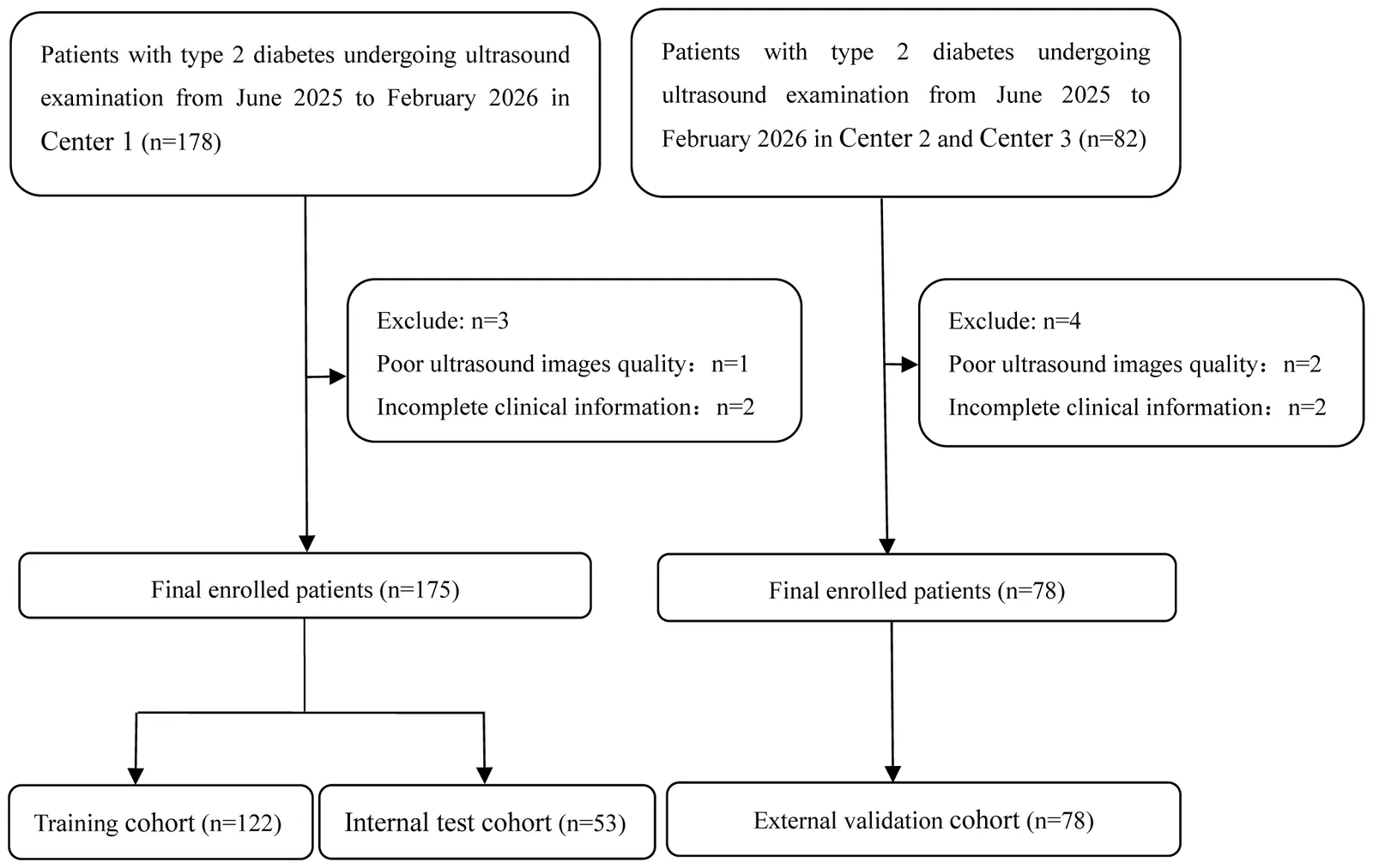
The patient recruitment flowchart.

**Figure 2:**
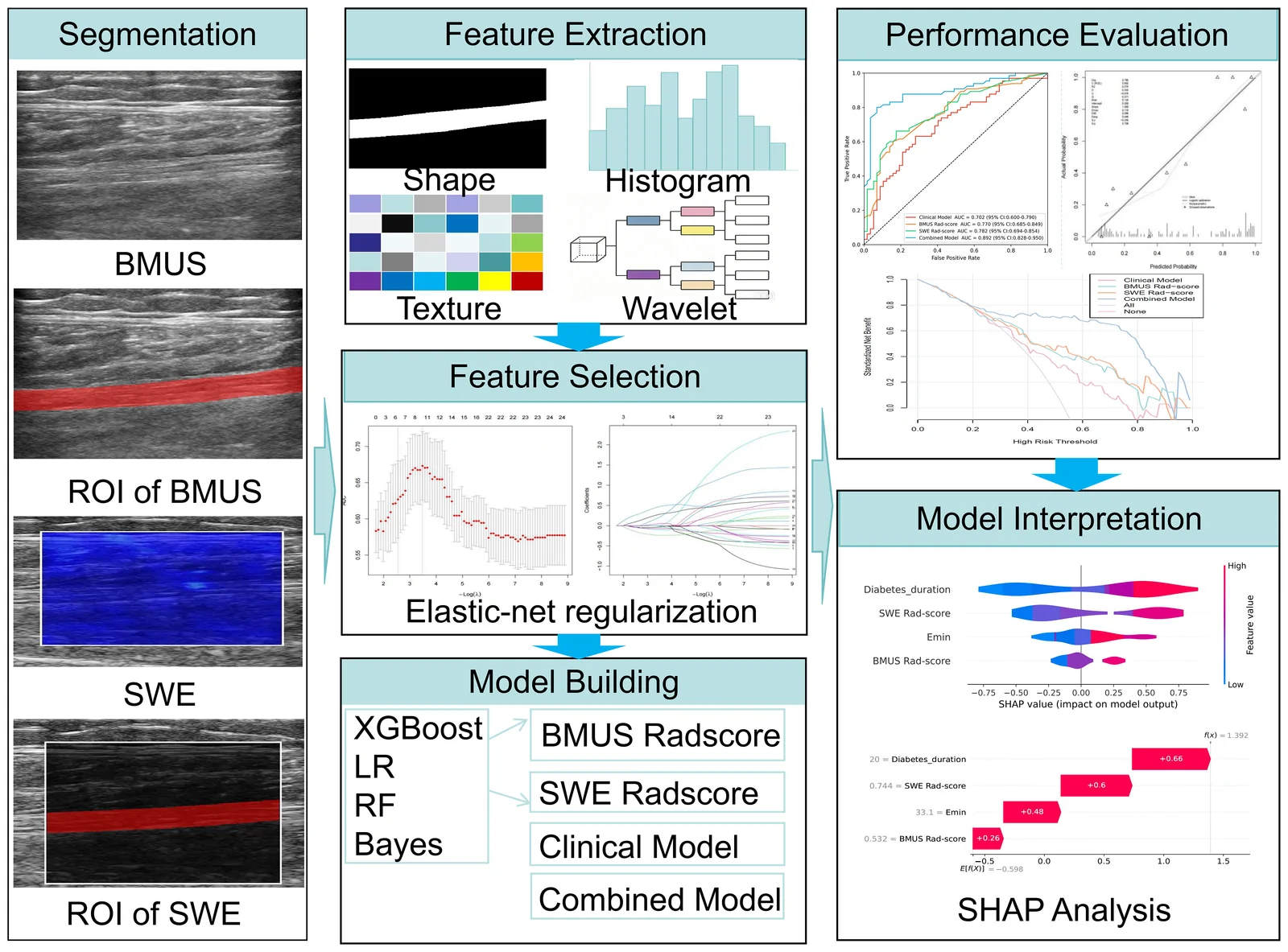
The overall study workflow. BMUS, B-mode ultrasound; LR, logistic regression; Rad-score, radiomics score; RF, random forest; ROI, region of interest; SHAP, SHapley Additive exPlanations; SWE, shear wave elastography; XGBoost, eXtreme Gradient Boosting.

After enrollment and provision of written informed consent, all participants completed a dedicated tibial nerve BMUS and SWE examination within two weeks (before or after) of their clinically indicated nerve conduction study. The ultrasound examination was performed as a study-specific research procedure and was not part of routine clinical care. A total of 253 patients were included in this study. Of these, 175 patients from Center 1 were randomly assigned to a training cohort (n = 122) and an internal test cohort (n = 53) in a 7:3 ratio, with the remaining 78 patients from Centers 2 and 3 serving as the external validation cohort. According to NCS findings, patients were categorized as either the DPN or non-DPN group. DPN was defined as conduction abnormalities detected in two or more peripheral nerves, following the American Diabetes Association diagnostic standards (5). NCS was performed bilaterally on the median, ulnar, tibial, common peroneal, superficial peroneal, and sural nerves. As NCS primarily assesses large myelinated nerve fiber function, the DPN cases identified in this study correspond to NCS-confirmed DPN. This study recruitment process and overall analytical workflow are depicted in **Figures 1**, **2**, respectively. Sample size justification was assessed *post-hoc* based on the events-per-variable (EPV) ratio. The training cohort contained 65 DPN events, and after rigorous dimensionality reduction, the final model retained 4 variables, yielding an EPV of 65/4 = 16.25, which exceeds the recommended minimum threshold of 10, supporting the stability of the regression estimates. Furthermore, model generalizability was independently evaluated in an external validation cohort of 78 patients from two geographically distinct centers, providing support for the adequacy of the current sample size.

### 2.2 Clinical data collection

At enrollment, clinical data were retrieved from electronic medical records, comprising age, sex, body mass index (BMI), hypertension history, glycated hemoglobin (HbA1c), diabetes duration, and NCS results.

### 2.3 Multimodal US image acquisition

At all three centers, BMUS and SWE imaging was performed using the Mindray Resona 7s system (Mindray Medical International) equipped with an L11–3 linear-array transducer (3–11 MHz). Three experienced ultrasonographers (> 8 years) conducted all examinations on the right lower extremity, blinded to patients’ clinical histories and electrophysiological results. Patients were placed supine with the ankle in slight plantar flexion and mild external rotation. Abundant coupling gel was applied and light transducer pressure was maintained throughout acquisition to avoid tissue compression artifacts.

BMUS was first performed with the transducer positioned transversely approximately 4 cm proximal to the medial malleolus, from which cross-sectional images of the tibial nerve (TN) were acquired to record the transverse diameter, anteroposterior diameter, perimeter, and cross-sectional area. The transducer was subsequently rotated 90°for optimal longitudinal B-mode imaging, in the same plane of which SWE images were obtained by the same operator using integrated SWE software. All SWE measurements were obtained with the transducer held stationary for several seconds to allow the elastography signal to stabilize. Within the elastography window, a 2 mm diameter circular region of interest (ROI) was placed, and the mean (Emean), minimum (Emin), and maximum (Emax) shear modulus values (kPa) were each derived from three repeated measurements. All images were reviewed at the time of acquisition and immediately reacquired if deemed diagnostically inadequate.

### 2.4 ROI segmentation

Manual segmentation of the TN on optimal BMUS and SWE longitudinal images was performed using ITK-SNAP (version 3.8.0; https://www.itksnap.org). The ROI was delineated along the hyperechoic medial border of the TN independently by two readers: Reader 1 (8 years of musculoskeletal ultrasound experience) performed segmentation for all 253 patients, and Reader 2 (5 years of experience) independently segmented a randomly selected subset of 30 patients to assess inter-observer reproducibility. Both readers were blinded to all clinical information. Reader 1 repeated segmentation for the same 30 patients after a two-week interval to evaluate intra-observer reproducibility. Features yielding an intraclass correlation coefficient (ICC) ≥ 0.75 were considered reproducible and retained for subsequent analysis.

### 2.5 Radiomics feature extraction and selection

Radiomics features were extracted from BMUS and SWE images using PyRadiomics (version 3.7) (18), which implements IBSI-compliant feature definitions, encompassing first-order statistics, shape, texture, and wavelet-transformed features. Prior to extraction, images were resampled to 1 × 1 × 1 mm^3^ isotropic voxels, normalized, and discretized with a fixed bin count of 25. To prevent information leakage, all feature selection was performed exclusively within the training cohort. First, ICC-based reproducibility filtering was applied to retain stable features (ICC ≥ 0.75). ComBat harmonization was then performed to correct for inter-center batch effects, with the training cohort designated as the reference batch (19). Subsequently, within the training cohort, univariate Wilcoxon rank-sum testing (*p* < 0.05) was applied to identify DPN-associated features, followed by Spearman’s correlation filtering to remove redundant features (coefficient ≥ 0.8), and finally elastic-net logistic regression to identify the optimal discriminative feature subset. The selected feature subset was then used for model development and validation across all three cohorts.

### 2.6 Radiomics model development and Rad-score construction

Using the selected features, four machine learning algorithms were trained to construct individual BMUS and SWE radiomics models: logistic regression (LR), random forest (RF), naive Bayes, and eXtreme Gradient Boosting (XGBoost). Optimal hyperparameters for each algorithm were determined through a combination of grid search and ten-fold cross-validation. Hyperparameter optimization for each algorithm was performed via grid search combined with ten-fold cross-validation. The algorithm demonstrating the highest discriminative performance across all three cohorts was adopted to derive modality-specific Rad-scores.

### 2.7 Combined model construction

To identify independently associated factors of DPN, both univariable and multivariable logistic regression analyses were conducted on clinical and conventional ultrasound variables. The resulting independent factors, together with the BMUS and SWE Rad-scores, were incorporated into an XGBoost-based combined diagnostic model. All performance metrics reported for the combined model are those of the XGBoost model. To facilitate clinical visualization, a logistic regression model was additionally fitted using the same set of features, and a nomogram was derived from its coefficients in the training cohort. For benchmarking purposes, a reference model comprising solely the independently associated clinical and conventional ultrasound variables was constructed in parallel.

### 2.8 Model performance evaluation

Model discrimination was quantified using area under the receiver operating characteristic curve (AUC), along with accuracy, sensitivity, and specificity, across all three cohorts. Calibration was examined through calibration plots and the Hosmer-Lemeshow test, with *p* > 0.05 denoting satisfactory agreement between estimated and observed outcomes. The Brier score was used to evaluate overall accuracy, with lower values reflecting better performance. Calibration slopes and intercepts were additionally reported. Net clinical benefit was assessed through decision curve analysis (DCA) over a spectrum of threshold probabilities (20).

### 2.9 Model interpretability analysis

To enhance model interpretability, the SHAP method was employed to quantify the contribution of each variable to the combined model’s output, based on cooperative game theory principles that decompose each model output into additive feature-level contributions (21). At the global level, a SHAP summary plot was generated to rank features by mean absolute SHAP values and characterize the directional relationship between feature magnitude and model output across all training samples. At the individual level, SHAP waterfall plots were generated for representative non-DPN and DPN cases to decompose each model output into sequential additive feature contributions, with the mean training cohort output serving as the baseline expected value [E(f(X))], thereby elucidating output heterogeneity across patients with varying risk profiles.

### 2.10 Statistical methods

All statistical analyses were conducted in Python (version 3.7) and R (version 4.3.1). Normally distributed continuous variables are expressed as mean ± standard deviation (SD), non-normally distributed variables as median (interquartile range), and categorical variables as frequencies and percentages. Between-group differences were evaluated using the Wilcoxon rank-sum test, Student’s t-test, chi-squared test, or Fisher’s exact test. Segmentation reproducibility was quantified using intraclass correlation coefficients (ICC ≥ 0.75). Model discrimination was assessed using ROC curves, AUC, accuracy, sensitivity, and specificity; between-model AUC differences were evaluated via the DeLong test. Calibration was evaluated using calibration curves, the Hosmer-Lemeshow test, Brier scores, and calibration slopes and intercepts. Clinical utility was assessed via decision curve analysis. Statistical significance was defined as a two-sided *p* < 0.05.

## 3 Results

### 3.1 Patient characteristics

Among 253 consecutive T2DM patients enrolled, DPN prevalence across the three cohorts was 53.3% (65/122) in training, 52.8% (28/53) in internal test, and 66.7% (52/78) in external validation. Demographic and clinical characteristics of all participants are presented in **Table 1**. Diabetes duration was the most consistently discriminating variable, with DPN patients showing markedly longer duration than non-DPN patients across all three cohorts (all *p* < 0.05). DPN patients were also older across the training and external validation cohorts (*p* < 0.05 for both). Hypertension history differed significantly between groups in the external validation cohort (*p* = 0.008), as did perimeter and CSA in the internal test cohort (*p* = 0.035 and *p* = 0.022, respectively). All remaining clinical and ultrasonographic parameters, including all three SWE elasticity indices, did not differ significantly between groups across cohorts (all *p* > 0.05).

**Table 1:** Baseline clinical and ultrasonographic characteristics in training, internal test, and external validation cohorts.

Variable	Statistic	Training (n = 122)			Internal test (n = 53)			External validation (n = 78)		
		Non-DPN (n=57)	DPN (n=65)	*p*	Non-DPN (n=25)	DPN (n=28)	*p*	Non-DPN (n=26)	DPN (n=52)	*p*
Sex	Female, n (%)	17 (29.8)	21 (32.3)	0.921	11 (44.0)	11 (39.3)	0.945	9 (34.6)	26 (50.0)	0.295
	Male, n (%)	40 (70.2)	44 (67.7)		14 (56.0)	17 (60.7)		17 (65.4)	26 (50.0)	
Age (years)	Mean ± SD	50.88 ± 15.99	56.28 ± 11.66	0.038	52.00 ± 14.96	56.46 ± 12.30	0.239	51.12 ± 12.89	57.81 ± 10.84	0.018
BMI (kg/m^2^)	Median (IQR)	24.84 (22.48–27.85)	23.73 (21.34–25.65)	0.074	22.22 (21.22–25.39)	22.68 (20.17–24.70)	0.587	26.24 (24.93–28.37)	26.15 (23.59–29.06)	0.596
Diabetes duration (years)	Median (IQR)	4.00 (1.00–10.00)	10.00 (6.00–15.00)	< 0.001	5.00 (1.00–7.00)	10.00 (4.50–16.50)	0.009	3.00 (1.00–8.00)	10.00 (6.00–14.00)	< 0.001
HbA1c (%)	Median (IQR)	9.00 (7.50–11.00)	8.60 (7.70–10.30)	0.953	8.30 (7.30–10.40)	8.85 (7.65–10.75)	0.318	9.00 (7.40–10.60)	8.50 (7.50–10.10)	0.508
Hypertension	Negative, n (%)	36 (63.2)	38 (58.5)	0.731	18 (72.0)	19 (67.9)	0.977	18 (69.2)	18 (34.6)	0.008
	Positive, n (%)	21 (36.8)	27 (41.5)		7 (28.0)	9 (32.1)		8 (30.8)	34 (65.4)	
Transverse diameter (cm)	Mean ± SD	0.49 ± 0.06	0.50 ± 0.06	0.329	0.48 ± 0.09	0.52 ± 0.08	0.103	0.54 ± 0.11	0.49 ± 0.13	0.066
Anteroposterior diameter (cm)	Median (IQR)	0.30 (0.27–0.34)	0.31 (0.27–0.35)	0.201	0.31 (0.28–0.34)	0.34 (0.29–0.35)	0.352	0.31 (0.26–0.36)	0.31 (0.24–0.38)	0.567
Perimeter (cm)	Mean ± SD	1.24 ± 0.16	1.27 ± 0.15	0.268	1.24 ± 0.16	1.34 ± 0.16	0.035	1.46 ± 0.26	1.38 ± 0.36	0.436
CSA (cm^2^)	Mean ± SD	0.10 ± 0.02	0.11 ± 0.02	0.121	0.10 ± 0.13	0.11 ± 0.02	0.022	0.12 ± 0.04	0.13 ± 0.05	0.781
Emean (kPa)	Median (IQR)	35.90 (29.10–44.47)	37.40 (31.07–47.57)	0.353	34.00 (26.50–43.90)	36.53 (31.34–48.95)	0.265	28.10 (19.50–38.80)	34.20 (26.85–40.10)	0.376
Emin (kPa)	Median (IQR)	28.40 (23.30–37.57)	31.95 (25.55–38.47)	0.234	27.90 (21.47–34.83)	30.50 (27.38–40.06)	0.176	24.18 (16.06–32.12)	27.30 (21.02–32.32)	0.388
Emax (kPa)	Median (IQR)	46.43 (34.93–53.70)	47.92 (38.10–60.70)	0.311	44.80 (35.10–52.40)	42.69 (36.19–59.84)	0.640	38.38 (30.37–58.35)	40.94 (31.73–50.83)	0.970

IQR, interquartile range; SD, standard deviation. Data are presented as mean ± SD, median (interquartile range), or n (%). P-values were derived from the independent samples t-test, Wilcoxon rank-sum test, or chi-squared test, as appropriate.

### 3.2 Radiomics feature extraction and selection

Per-patient feature extraction from BMUS and SWE images produced 1,888 radiomics features in total (944 per modality). Following ICC filtering and ComBat harmonization, Wilcoxon testing identified 73 BMUS and 95 SWE DPN-associated features; Spearman filtering reduced these to 10 BMUS and 24 SWE candidates; elastic-net selection yielded 8 BMUS and 10 SWE final features, predominantly encompassing first-order, texture, and wavelet-transformed descriptors. For BMUS, 8 features were retained: one first-order feature (Range), one gray-level run-length matrix (GLRLM) feature (LongRunLowGrayLevelEmphasis), two wavelet-LHL gray-level size zone matrix (GLSZM) features (GrayLevelNonUniformityNormalized and SmallAreaLowGrayLevelEmphasis), one wavelet-LHH gray-level co-occurrence matrix (GLCM) feature (SumEntropy), one wavelet-LHH GLSZM feature (LargeAreaHighGrayLevelEmphasis), one wavelet-HLH first-order feature (Entropy), and one wavelet-HHH GLSZM feature (GrayLevelNonUniformity). For SWE, 10 features were retained: one shape feature (MajorAxisLength), one gray-level dependence matrix (GLDM) feature (DependenceVariance), one original GLSZM feature (SmallAreaEmphasis), one wavelet-LLH GLRLM feature (LongRunLowGrayLevelEmphasis), one wavelet-LLH GLSZM feature (ZonePercentage), one wavelet-HLL GLSZM feature (SizeZoneNonUniformityNormalized), one wavelet-HLH GLSZM feature (SmallAreaHighGrayLevelEmphasis), one wavelet-HHH GLRLM feature (RunPercentage), one wavelet-LLL first-order feature (RobustMeanAbsoluteDeviation), and one wavelet-LLL neighborhood gray-tone difference matrix (NGTDM) feature (Busyness).

### 3.3 Radiomics model performance and Rad-score construction

Four classifiers — logistic regression (LR), random forest (RF), naive Bayes, and XGBoost — were each trained on the final radiomics feature subset for both BMUS and SWE modalities, with performance metrics summarized in **Tables 2**, **3**. XGBoost consistently outperformed the other algorithms for both BMUS and SWE modalities across all three cohorts. In the BMUS modality, the XGBoost model yielded AUC values of 0.770 (95% CI: 0.685–0.849), 0.702 (95% CI: 0.547–0.833), and 0.715 (95% CI: 0.578–0.838) in the training, internal test, and external validation cohorts, respectively; the corresponding values for the SWE modality were 0.782 (95% CI: 0.694–0.854), 0.736 (95% CI: 0.595–0.871), and 0.726 (95% CI: 0.615–0.839). The predicted probabilities derived from the BMUS and SWE XGBoost models were therefore designated as the BMUS Rad-score and SWE Rad-score, respectively, and subsequently used as inputs for combined modeling.

**Table 2:** Performance of four machine learning models based on BMUS radiomics features for classifying DPN across training, internal test, and external validation cohorts.

Cohort	Model	AUC (95% CI)	Accuracy	Sensitivity	Specificity	Cutoff
Training	XGBoost	0.770 (0.685–0.849)	0.713	0.600	0.842	0.403
	LR	0.702 (0.612–0.791)	0.664	0.523	0.825	0.597
	RF	0.749 (0.665–0.830)	0.705	0.662	0.754	0.554
	Naive Bayes	0.701 (0.605–0.790)	0.672	0.523	0.842	0.880
Internal test	XGBoost	0.702 (0.547–0.833)	0.660	0.536	0.800	0.403
	LR	0.649 (0.496–0.784)	0.585	0.357	0.840	0.597
	RF	0.611 (0.442–0.762)	0.604	0.500	0.720	0.554
	Naive Bayes	0.620 (0.443–0.767)	0.642	0.429	0.880	0.880
External validation	XGBoost	0.715 (0.578–0.838)	0.654	0.596	0.769	0.403
	LR	0.656 (0.525–0.784)	0.513	0.404	0.731	0.597
	RF	0.695 (0.560–0.821)	0.474	0.308	0.808	0.554
	Naive Bayes	0.604 (0.483–0.728)	0.449	0.192	0.962	0.880

XGBoost, eXtreme Gradient Boosting; LR, logistic regression; RF, random forest; Naive Bayes, Gaussian naïve Bayes classifier. AUC values are presented with 95% confidence intervals.

**Table 3:** Performance of four machine learning models based on SWE radiomics features for classifying DPN across training, internal test, and external validation cohorts.

Cohort	Model	AUC (95% CI)	Accuracy	Sensitivity	Specificity	Cutoff
Training	XGBoost	0.782 (0.694–0.854)	0.738	0.662	0.825	0.382
	LR	0.759 (0.677–0.840)	0.730	0.785	0.667	0.479
	RF	0.731 (0.636–0.816)	0.770	0.585	0.982	0.572
	Naive Bayes	0.770 (0.687–0.849)	0.754	0.785	0.719	0.210
Internal test	XGBoost	0.736 (0.595–0.871)	0.736	0.679	0.800	0.382
	LR	0.691 (0.540–0.830)	0.660	0.750	0.560	0.479
	RF	0.604 (0.447–0.761)	0.547	0.536	0.560	0.572
	Naive Bayes	0.659 (0.509–0.794)	0.642	0.750	0.520	0.210
External validation	XGBoost	0.726 (0.615–0.839)	0.577	0.442	0.846	0.382
	LR	0.650 (0.521–0.779)	0.679	0.769	0.500	0.479
	RF	0.699 (0.579–0.815)	0.628	0.654	0.577	0.572
	Naive Bayes	0.713 (0.592–0.840)	0.667	0.808	0.385	0.210

XGBoost, eXtreme Gradient Boosting; LR, logistic regression; RF, random forest; Naive Bayes, Gaussian naïve Bayes classifier. AUC values are presented with 95% confidence intervals.

### 3.4 Clinical model performance

As shown in **Table 4**, univariable and multivariable logistic regression analyses identified diabetes duration (OR = 1.09, 95% CI: 1.03–1.16, *p* = 0.003) and Emin (OR = 1.04, 95% CI: 1.01–1.07, *p* = 0.020) as independently associated factors of DPN. Based on these two variables, the clinical model achieved AUC values of 0.702 (95% CI: 0.600–0.790), 0.721 (95% CI: 0.579–0.843) and 0.759 (95% CI: 0.634–0.874) in the training, internal test, and external validation cohorts, respectively.

**Table 4:** Univariable and multivariable logistic regression analyses based on clinical and ultrasonographic variables.

Variable	Level	Univariable logistic analysis		Multivariable logistic analysis	
		OR (95% CI)	*p*	OR (95% CI)	*p*
Sex	Female (ref)	—		—	
	Male	1.07 (0.52–2.23)	0.852	—	
Age (years)		1.02 (1.00–1.05)	0.084	—	
BMI (kg/m^2^)		0.91 (0.83–1.01)	0.068	—	
Diabetes duration (years)		1.08 (1.02–1.14)	0.007	1.09 (1.03–1.16)	0.003
HbA1c (%)		0.99 (0.84–1.16)	0.912	—	
Hypertension history	Negative (ref)	—		—	
	Positive	1.21 (0.60–2.46)	0.590	—	
Transverse diameter (cm)		13.74 (0.06–3314.03)	0.349	—	
Anteroposterior diameter (cm)		1.07 (1.00–1.16)	0.060	—	
Perimeter (cm)		4.38 (0.42–45.70)	0.217	—	
CSA (cm^2^)		1.15 (0.99–1.33)	0.071	—	
Emean (kPa)		1.02 (1.00–1.04)	0.072	—	
Emin (kPa)		1.03 (1.00–1.06)	0.047	1.04 (1.01–1.07)	0.020
Emax (kPa)		1.01 (1.00–1.02)	0.130	—	

OR, odds ratio; CI, confidence interval. —, not included in the multivariable model. Reference categories for categorical variables are indicated by "ref". p-values were derived from logistic regression.

### 3.5 Performance of the combined model

**Figure 3:**
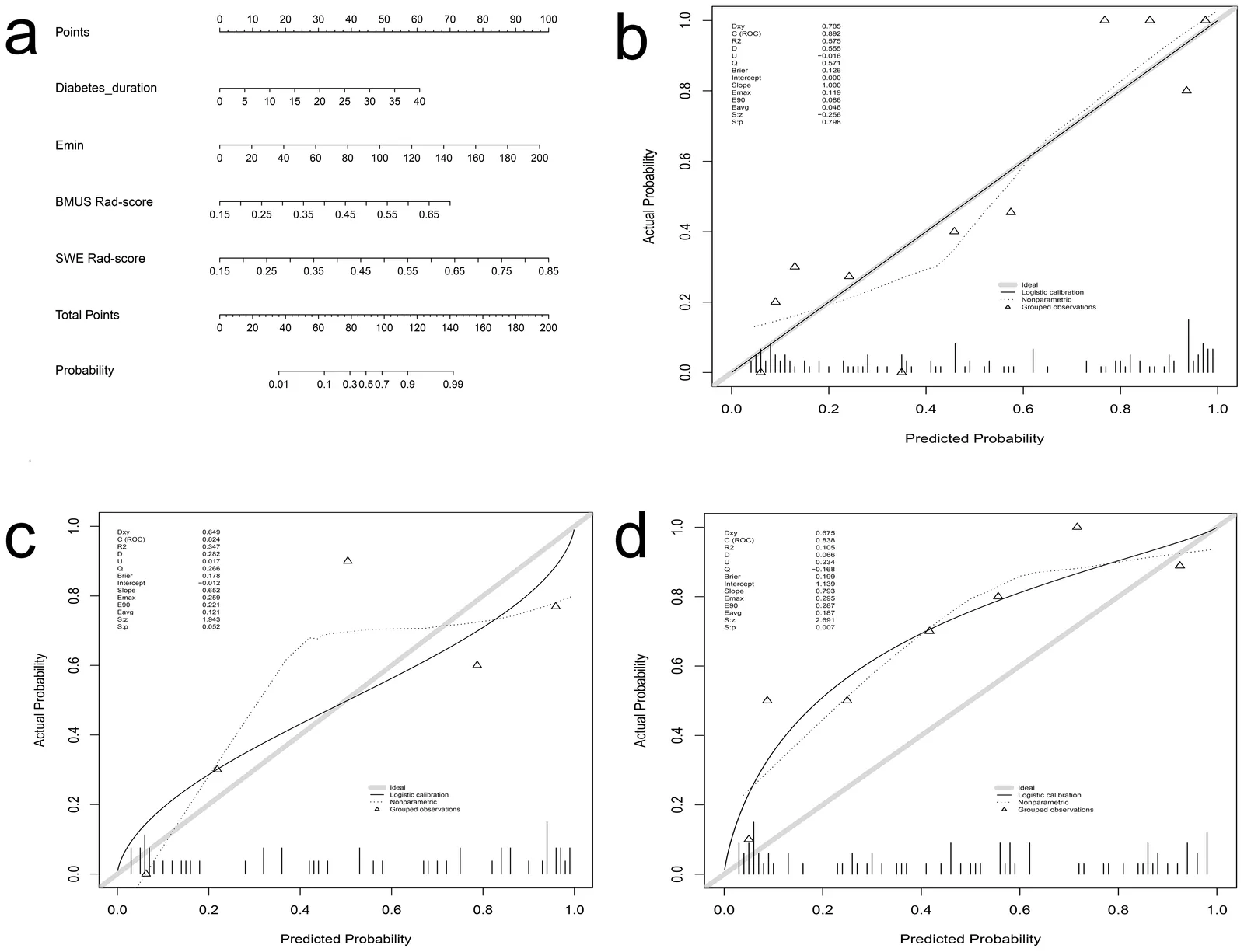
Nomogram for clinical visualization and calibration curves of the combined model for diabetic peripheral neuropathy (DPN) classification. **(a)** Nomogram for clinical visualization, incorporating diabetes duration, minimum elastic modulus (Emin), B-mode ultrasound (BMUS) Rad-score, and shear wave elastography (SWE) Rad-score. **(b–d)** Calibration curves of the XGBoost combined model in the training **(b)**, internal test **(c)**, and external validation **(d)** cohorts. The diagonal gray line indicates perfect calibration, the solid black line denotes logistic calibration, and the dashed black line reflects non-parametric calibration. The nomogram serves as a visualization tool; all reported performance metrics are those of the XGBoost model.

The BMUS Rad-score, SWE Rad-score, diabetes duration, and Emin were incorporated into an XGBoost-based combined diagnostic model and presented as a nomogram for individualized DPN risk estimation (**Figure 3a**). Calibration curves demonstrated good agreement between estimated and observed DPN probabilities in both the training and internal test cohorts (*p* > 0.05; **Figures 3b, c**). In the external validation cohort, the combined model maintained discriminative performance (AUC = 0.838), while calibration showed statistically significant deviation (*p* < 0.05; **Figure 3d**). Brier scores were 0.126, 0.178, and 0.199 in the training, internal test, and external validation cohorts, respectively. Calibration slopes were 1.000, 0.652, and 0.793, and calibration intercepts were 0, −0.012, and 1.139 in the training, internal test, and external validation cohorts, respectively.

The combined model achieved the highest diagnostic performance across all cohorts. In the training cohort, it attained an AUC of 0.892 (95% CI: 0.828–0.950), with an accuracy of 86.1%, sensitivity of 80.0%, and specificity of 93.0%. In the internal test cohort, the AUC reached 0.824 (95% CI: 0.696–0.925), with an accuracy of 67.9%, sensitivity of 64.3%, and specificity of 72.0%. In the external validation cohort, the combined model achieved an AUC of 0.838 (95% CI: 0.735–0.926), accuracy of 67.9%, sensitivity of 55.8%, and specificity of 92.3% (**Table 5**).

**Table 5:** Diagnostic performance of four models for DPN across training, internal test, and external validation cohorts.

Cohort	Model	AUC (95% CI)	Accuracy	Sensitivity	Specificity	Cutoff
Training	Clinical model	0.702 (0.600–0.790)	0.672	0.631	0.719	0.529
	BMUS Rad-score	0.770 (0.685–0.849)	0.713	0.600	0.842	0.403
	SWE Rad-score	0.782 (0.694–0.854)	0.738	0.662	0.825	0.382
	Combined model	0.892 (0.828–0.950)	0.861	0.800	0.930	0.400
Internal test	Clinical model	0.721 (0.579–0.843)	0.698	0.679	0.720	0.529
	BMUS Rad-score	0.702 (0.547–0.833)	0.660	0.536	0.800	0.403
	SWE Rad-score	0.736 (0.595–0.871)	0.736	0.679	0.800	0.382
	Combined model	0.824 (0.696–0.925)	0.679	0.643	0.720	0.400
External validation	Clinical model	0.759 (0.634–0.874)	0.615	0.500	0.846	0.529
	BMUS Rad-score	0.715 (0.578–0.838)	0.654	0.596	0.769	0.403
	SWE Rad-score	0.726 (0.615–0.839)	0.577	0.442	0.846	0.382
	Combined model	0.838 (0.735–0.926)	0.679	0.558	0.923	0.400

AUC values are presented with 95% confidence intervals.

### 3.6 Model comparison

**Figure 4:**
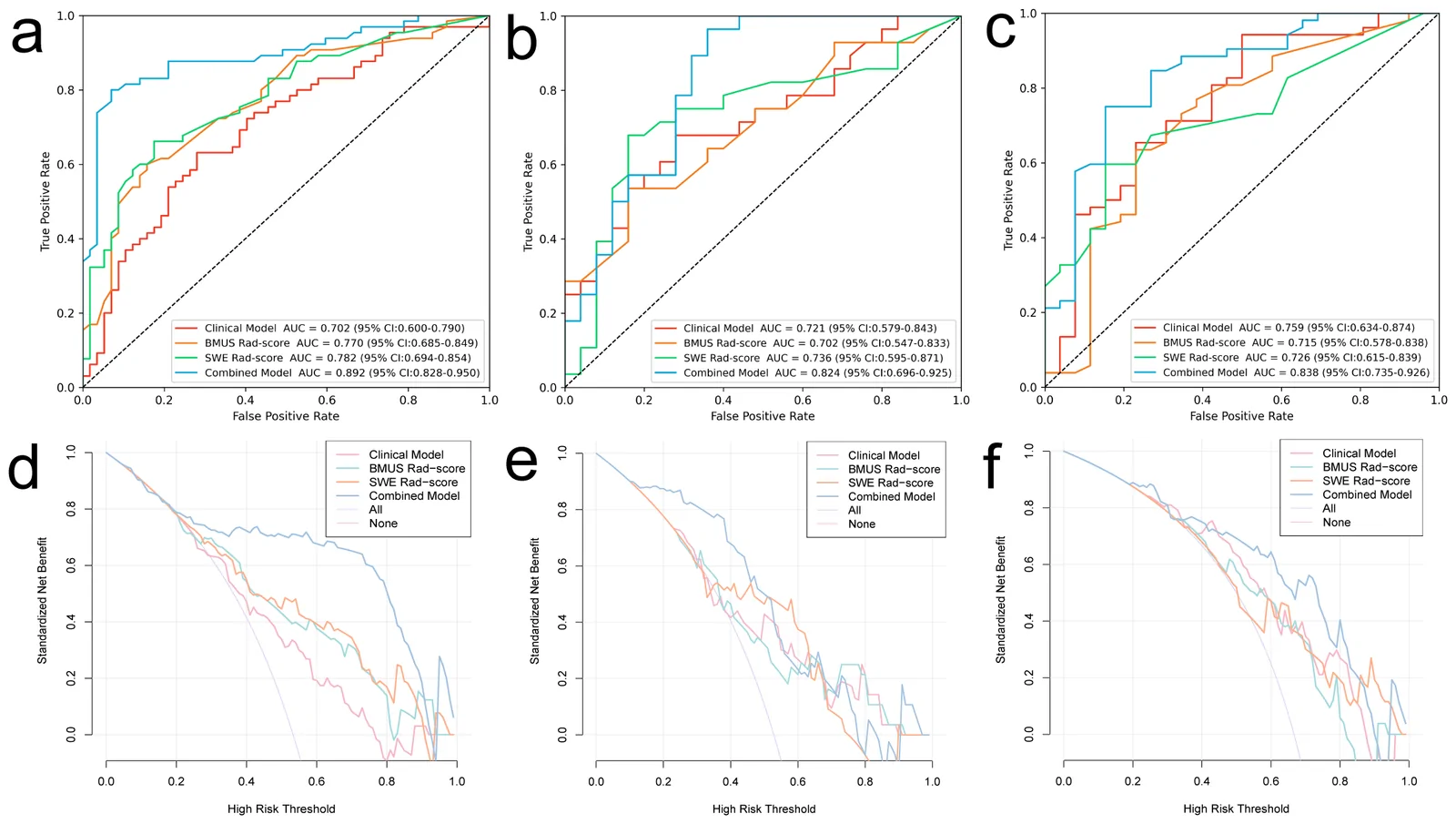
Receiver operating characteristic (ROC) curves and decision curve analysis (DCA) of four models across three cohorts. **(a–c)** ROC curves for the clinical model, B-mode ultrasound (BMUS) Rad-score, shear wave elastography (SWE) Rad-score, and combined model in the training **(a)**, internal test **(b)**, and external validation **(c)** cohorts. The area under the curve (AUC) with 95% confidence interval (CI) is indicated for each model. **(d–f)** DCA curves in the training **(d)**, internal test **(e)**, and external validation **(f)** cohorts.

**Table 5** summarizes the performance metrics of all four models across the three cohorts. The combined model significantly outperformed each individual model in the training cohort (AUCs: 0.892 vs. 0.782, 0.770, and 0.702 for SWE Rad-score, BMUS Rad-score, and clinical model, respectively; all *p* < 0.05), internal test cohort (AUCs: 0.824 vs. 0.736, 0.702, and 0.721; all *p* < 0.05), and external validation cohort (AUCs: 0.838 vs. 0.726, 0.715, and 0.759; all *p* < 0.05) (**Figures 4a–c**). Decision curve analysis demonstrated that the combined model provided greater net clinical benefit than the clinical model, BMUS Rad-score, and SWE Rad-score across most threshold probabilities in all three cohorts (**Figures 4d–f**).

### 3.7 Model interpretability analysis

**Figure 5:**
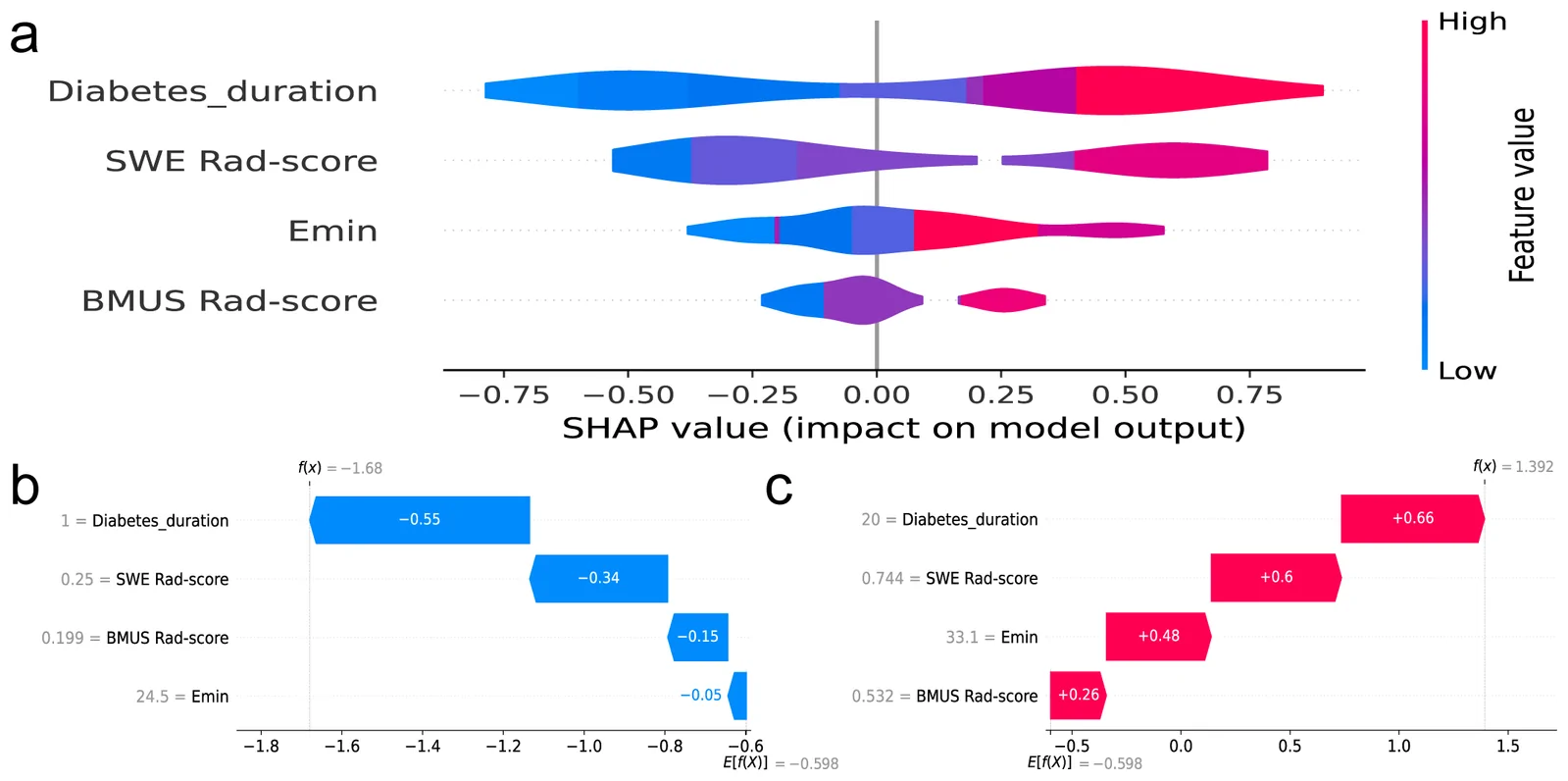
SHAP-based interpretability analysis of the combined model. **(a)** Global SHAP summary plot ranking features by mean absolute SHAP value; each point represents one patient, with color indicating feature value from low **(blue)** to high (red); positive and negative SHAP values indicate increased and decreased estimated DPN probability, respectively. **(b)** SHAP waterfall plot for a representative non-DPN case; all four features exhibited negative contributions. **(c)** SHAP waterfall plot for a representative DPN case; all four features exhibited positive contributions. The baseline expected value [E(f(X))] was −0.598 for both cases. BMUS, B-mode ultrasound; Emin, minimum elastic modulus; SHAP, SHapley Additive exPlanations; SWE, shear wave elastography.

To enhance model transparency, SHAP decomposition was applied to elucidate the contribution of each variable to the combined model’s output. At the global scale, the beeswarm plot (**Figure 5a**) revealed that diabetes duration exerted the greatest influence on DPN classification, followed by SWE Rad-score, Emin, and BMUS Rad-score, ranked by their mean absolute SHAP values; across all four variables, higher feature values corresponded consistently to greater positive model output contributions. Individual-level interpretability was further demonstrated using SHAP waterfall plots for two representative cases, with the mean model output of the training cohort as the baseline expected value [E(f(X)) = −0.598]. For the DPN-negative case (**Figure 5b**), all four features exhibited negative SHAP contributions, with diabetes duration (1 year, SHAP = −0.55) and SWE Rad-score (0.250, SHAP = −0.34) exerting the greatest negative influence, yielding a low model output (f(x) = −1.68) concordant with the NCS-negative finding. For the DPN-positive case (**Figure 5c**), all four features exhibited positive contributions, with diabetes duration (20 years, SHAP = +0.66) and SWE Rad-score (0.744, SHAP = +0.60) exerting the greatest positive influence, yielding a high model output (f(x) = 1.392) consistent with the NCS-confirmed DPN diagnosis. The individual-level SHAP feature importance ranking was consistent with the global ranking, with diabetes duration and SWE Rad-score ranking highest in both analyses.

## 4 Discussion

This multicenter prospective cross-sectional diagnostic study demonstrated that a dual-modality radiomics model integrating the SWE Rad-score, BMUS Rad-score, diabetes duration, and Emin consistently outperformed all other models across three cohorts, yielding AUC values of 0.892, 0.824, and 0.838 in the training, internal test, and external validation sets, respectively. Among the four machine learning algorithms evaluated, XGBoost exhibited the strongest discriminative performance and was therefore adopted to generate the Rad-scores. The model extends beyond conventional morphometric and single-point elastographic assessments, offering a practical complement to nerve conduction studies, particularly where electrophysiological assessment is unavailable.

The BMUS radiomics features selected in the present study share notable commonalities with prior ultrasound radiomics investigations of DPN, with entropy-related and gray-level heterogeneity descriptors predominating, findings consistent with the echo intensity disorganization caused by progressive demyelination and axonal degeneration documented in diabetic nerve tissue (5). Byra et al. further validated this association by demonstrating significant correlations between B-mode image entropy and combined collagen and myelin concentrations within nerve fascicles (R = −0.51), directly linking these texture features to myelinated fiber integrity (22). The predominance of multi-scale stiffness heterogeneity features among the selected SWE radiomics descriptors suggests that the biomechanical microarchitecture of the tibial nerve, rather than its macroscopic mean stiffness, encodes the most discriminative information for DPN classification (8). This complementarity is further supported by the absence of inter-modality feature redundancy following Spearman correlation filtering, confirming that BMUS and SWE radiomics encoded statistically independent dimensions of tibial nerve pathology and providing the methodological basis for their integration within the combined model.

Building upon these complementary feature sets, XGBoost demonstrated the strongest discriminative performance in both BMUS and SWE radiomics models, suggesting that its capacity to model complex nonlinear relationships among imaging biomarkers confers particular advantage in high-dimensional radiomics settings (23). Among prior ultrasound radiomics investigations of DPN, Jiang et al. constructed a co-plane radiomics diagnostic model integrating transverse and longitudinal gray-scale BMUS features from 262 patients, achieving an external AUC of 0.70 (15); however, their model did not incorporate SWE-derived biomechanical features, limiting characterization to the structural echo domain alone. To the best of our knowledge, the present study is the first to integrate high-dimensional SWE radiomics with BMUS radiomics, quantitative elasticity indices, and clinical variables within a unified dual-modality model framework for DPN classification, offering a more comprehensive characterization of tibial nerve pathology than any prior single-modality approach.

Diabetes duration and Emin were independently associated with DPN on multivariable logistic regression (*p* = 0.003 and *p* = 0.020, respectively). The association with diabetes duration is consistent with a meta-analysis of 12,116 diabetic patients confirming significantly increased DPN risk with longer disease duration (24), and with a nationwide cross-sectional study of 14,908 T2DM patients across 24 Chinese provinces (25). Mechanistically, prolonged hyperglycemia drives oxidative stress, advanced glycation end-product accumulation, and endoneurial microvascular ischemia, collectively accelerating axonal degeneration and myelin loss (26). Regarding Emin, Jiang et al. demonstrated its good diagnostic accuracy for DPN, outperforming Emax (27). The present study extends these findings by confirming that Emin retains independent diagnostic value after covariate adjustment, suggesting that focal zones of minimum nerve stiffness reflect early endoneurial edema or localized demyelination preceding diffuse nerve stiffening. The non-significance of Emean and Emax likely reflects greater susceptibility to inter-patient variability and measurement noise. The clinical model achieved acceptable discriminative performance, with AUC values of 0.702, 0.721, and 0.759 across the three cohorts.

The integration of dual-modality radiomics scores with clinical variables yielded further performance gains beyond any individual component. Adequate calibration was confirmed in the training and internal test cohorts (both *p* > 0.05); however, calibration in the external validation cohort was suboptimal (*p* < 0.05). The Brier score in this cohort (0.199) remained below the naïve baseline of approximately 0.22, suggesting the miscalibration is moderate. This calibration drift may reflect a combination of differing DPN prevalence between cohorts (66.7% vs. 53.3%), residual inter-center heterogeneity not fully resolved by ComBat harmonization, and limited external sample size (n = 78) (28). The higher DPN prevalence in the external cohort likely reflects center-specific referral patterns. The calibration slopes of 0.652 (internal test) and 0.793 (external validation) indicate that predicted probabilities were more extreme than observed risk, consistent with moderate overfitting. We acknowledge that these slope values remain below the ideal, representing a limitation of the current model. Of note, decision curve analysis demonstrated consistent clinical benefit across all three cohorts, suggesting that the model retains clinical utility for risk stratification despite imperfect calibration. The preserved discriminative performance across cohorts (AUC 0.838) suggests that spectrum bias did not materially compromise risk ranking, although transportability to populations with substantially different DPN prevalence warrants further study. Wu et al. constructed a DPN nomogram integrating high-frequency ultrasound-derived CSA and SWE-derived shear wave velocity with clinical variables in 118 T2DM patients, achieving comparable diagnostic performance (29); however, their approach relied on conventional scalar measurements capturing only a limited sampling of nerve spatial heterogeneity, whereas the present study extracted high-dimensional radiomics features encoding multiscale texture and biomechanical complexity. Chen et al. reported higher AUC values (0.947, 0.910, and 0.887) in a larger multicenter cohort (14), likely attributable to a larger training sample and less stringent feature selection without inter-center batch correction. Beyond these methodological differences, Chen et al. relied exclusively on transverse-plane gray-scale BMUS radiomics without SWE-derived biomechanical characterization. In contrast, the present study applied ICC filtering, ComBat harmonization, and elastic-net regression to prioritize cross-center generalizable features, and additionally incorporated SWE radiomics features and quantitative elasticity indices, extending characterization into the biomechanical domain inaccessible from morphological imaging alone. The variability in external validation performance across studies—ranging from approximately 0.70 in Jiang et al. to 0.947 in Chen et al.—highlights the influence of differences in sample composition, imaging protocols, and feature selection strategies. It should also be acknowledged that published studies in this field remain limited and predominantly positive; independent replication by investigators unaffiliated with the original model-development centers is needed to establish a more balanced evidence base.

In the present study, the combined model achieved a specificity of 92.3% and a sensitivity of 55.8% in the external validation cohort. This profile—high specificity with a low false-positive rate—suggests that the model may be best suited as a rule-in adjunct for DPN: a positive result could help prioritize patients for confirmatory nerve conduction testing, while a negative result alone may not reliably exclude DPN. Clinical judgment therefore remains important in interpreting model outputs.

SHAP analysis illuminated the combined model’s decision-making at both global and individual levels (30). Diabetes duration emerged as the most influential variable, consistent with its role as the primary driver of cumulative peripheral nerve damage (25, 26). SWE Rad-score ranked second; its superiority over Emin supports the added discriminative value of multi-scale stiffness radiomics over single-point elastographic measurements (8). The directional consistency between SHAP-derived feature effects and established clinical knowledge supports the biological plausibility of the model’s learned decision rules (31). At the individual level, waterfall plots corroborated the global hierarchy: short diabetes duration and low SWE Rad-score drove DPN-negative classifications, while long diabetes duration and high SWE Rad-score drove DPN-positive classifications, with SHAP magnitudes consistent with global importance rankings. This interpretability framework may facilitate clinician understanding and support stratified intervention planning across patients with varying DPN risk profiles.

NCS, the reference standard in this study, detects dysfunction of large myelinated nerve fibers. Because DPN affects small fibers before large fibers, NCS-confirmed DPN reflects large-fiber involvement; in the absence of systematic clinical phenotyping, however, subclinical and confirmed DPN cannot be distinguished under the Toronto consensus criteria, and the present model accordingly identifies DPN at this NCS-confirmed level. In many primary care and resource-limited settings, NCS remains unavailable, and a substantial proportion of patients with established large-fiber DPN go undiagnosed—without receiving glycemic optimization, foot care education, or podiatric surveillance. The proposed ultrasound-based model, relying on equipment increasingly accessible in community-level hospitals, may serve as a noninvasive triage tool to identify these patients and prompt confirmatory referral. Earlier detection of existing but undiagnosed neuropathy can still avert irreversible complications such as foot ulceration. The model is not designed to replace NCS but to extend diagnostic capacity where electrophysiological assessment is unavailable. Future studies employing small-fiber function tests (e.g., corneal confocal microscopy, skin biopsy) as reference standards may enable detection at an earlier, subclinical stage.

The present study carries certain limitations. First, the modest sample size may restrict the generalizability of findings; validation in larger prospective multicenter cohorts is warranted. Second, several categories of data were not systematically collected: clinical phenotyping (neuropathy symptom scores, monofilament testing, vibration perception thresholds), preventing stratification by Toronto consensus criteria into subclinical versus confirmed DPN; potential confounders including height, renal function, vitamin B12 status, alcohol consumption, smoking history, peripheral arterial disease, and neurotoxic medication use; and age, which differed significantly between groups and is collinear with diabetes duration. In addition, hypertension prevalence differed substantially between cohorts, reflecting case-mix heterogeneity. These unmeasured factors may contribute to residual confounding and calibration drift. Third, calibration slopes in both the internal test and external validation cohorts deviated from the ideal, indicating moderate overfitting, and the external intercept reflected systematic risk underestimation attributable to differing DPN prevalence. Local recalibration is advisable before applying the model in new clinical settings. Fourth, sensitivity in the external validation cohort was moderate (55.8%), indicating that the model may miss a substantial proportion of DPN cases. Future studies should explore threshold optimization to improve sensitivity for screening applications. Fifth, manual ROI delineation may introduce inter-observer variability; deep learning-based automated segmentation should be explored to optimize the radiomics workflow. Sixth, this study was conducted exclusively in Chinese patients with T2DM; given potential ethnic differences in body habitus, nerve morphology, and DPN prevalence, external validation in diverse populations is warranted.

## 5 Conclusion

In conclusion, the proposed dual-modality ultrasound radiomics model integrating BMUS and SWE features with diabetes duration and Emin demonstrates favorable diagnostic performance in individualized DPN risk stratification. SHAP-based interpretability further supports transparent clinical decision-making. However, prospective validation in larger, geographically diverse cohorts is required before clinical deployment can be considered.

## Data Availability

The datasets used and analyzed during the current study are available from the corresponding author on reasonable request.
